# A Case of Angioimmunoblastic T-Cell Lymphoma and the Difficulties of Diagnosis

**DOI:** 10.7759/cureus.44566

**Published:** 2023-09-02

**Authors:** Michelle Koifman, Waqqas Tai, Daniel Castro, Amith Ahluwalia, Yingxian Liu

**Affiliations:** 1 Internal Medicine, The Brooklyn Hospital Center, Brooklyn, USA; 2 Hemotolgy and Oncology, The Brooklyn Hospital Center, Brooklyn, USA; 3 Hematology and Oncology, The Brooklyn Hospital Center, Brooklyn, USA; 4 Pathology, The Brooklyn Hospital Center, Brooklyn, USA

**Keywords:** malaria, chop, axillary lymphadenopathy, non-hodgkin lymphoma (nhl), aitl

## Abstract

Angioimmunoblastic T-cell lymphoma (AITL) is a rare and aggressive form of peripheral T-cell lymphoma (PTCL). It can present with signs and symptoms that have broad differentials, including fevers, night sweats, and skin rashes. In this case report, we present an interesting case of a young male of Nigerian descent with recently treated malaria who presented with such symptoms and a picture that was complicated, due to an inconclusive excisional biopsy for lymphoma. He was later diagnosed with AITL. Given the patient’s recent exposure to malaria, we will discuss the potential role malaria has in the development of AITL.

## Introduction

Angioimmunoblastic T-cell lymphoma (AITL) is a rare subtype of mature peripheral T-cell lymphoma (PTCL), a subtype of non-Hodgkin lymphoma (NHL). AITL derives from follicular helper T-cells and features a broad spectrum of clinical and pathological manifestations. It can present with generalized lymphadenopathy, fevers, skin rash, weight loss, hepatomegaly, splenomegaly, and autoimmune features, and can often be challenging to diagnose. AITL follows an aggressive clinical course, and patients may suffer from many infectious complications. Five-year overall survival (OS) rates range from 25% to 41%. Treatment options include steroids, combination chemotherapy, single-agent cytotoxic drugs, or novel agents such as alemtuzumab [1-13]. Infectious diseases such as malaria may also increase the risk of lymphoid neoplasms and lead to the worsening of OS through immunosuppression with cytokines such as interleukin-10 (IL-10) [14-17]. 

## Case presentation

**Figure 1:**
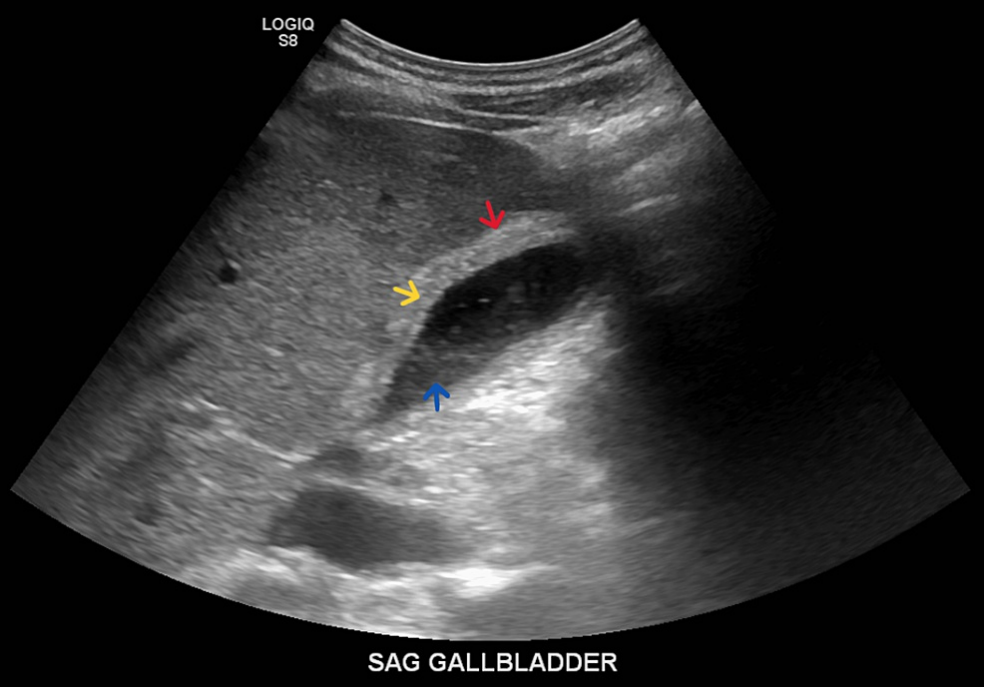
Right upper quadrant abdominal ultrasound showing gallbladder containing sludge (blue arrow), with gallbladder wall thickening (red arrow) and pericholecystic fluid (yellow arrow), suggestive of acute cholecystitis.

**Figure 2:**
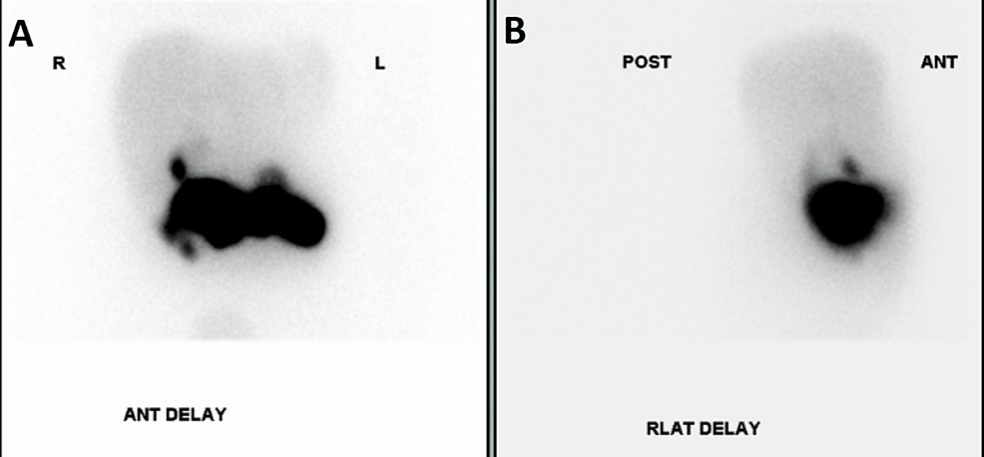
Hepatobiliary iminodiacetic acid (HIDA) scan showing patent common hepatic and common bile ducts; negative for extrahepatic biliary obstruction. Visualization (delayed) of the gallbladder which is negative for acute cholecystitis.

**Figure 3:**
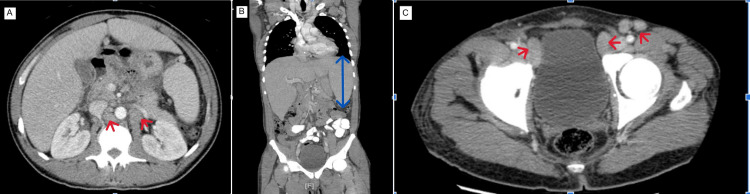
(A) Computed Tomography (CT) chest/abdomen/pelvis with contrast (axial view) showing retroperitoneal lymphadenopathy (red arrows). (B) CT chest/abdomen/pelvis with contrast (coronal view) showing splenomegaly (blue straight line with double arrows). (C) CT chest/abdomen/pelvis with contrast (axial view) showing pelvic and inguinal lymphadenopathy (red arrows).

**Figure 4:**
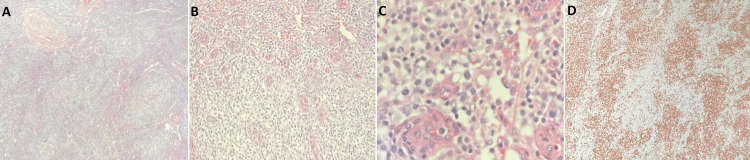
(A) Pathology of left inguinal lymph node under 40x magnification showing effacement of follicular pattern. (B) Pathology of left inguinal lymph node under 100x magnification showing prominent vessels surrounded by sheets of lymphoma cells. (C) Pathology of left inguinal lymph node under 400x magnification showing immunoblasts. (D) CD3 stain. Pathology of left inguinal lymph node under 40x magnification showing sheets of lymphoma cells.

We present the case of a 44-year-old male of Nigerian descent with a history of sickle cell trait and recently treated malaria infection about two weeks prior who presented to the hospital with fatigue, fever, malaise, chills, rigors, and loss of appetite for one month. The patient was tachycardic and with a fever of 102.3 F. Scleral icterus, epigastric tenderness on palpation, hepatosplenomegaly, and palpable inguinal lymphadenopathy were appreciated on physical exam. Initial laboratory studies showed anemia, elevated liver enzymes, and lactic acidosis. Other laboratory findings for the patient are documented in Table 1 below. The patient was treated for sepsis with fluids, ceftriaxone, and a dose of metronidazole. On admission, he was continued on ceftriaxone. The initial right upper quadrant abdominal ultrasound had findings suggestive of acute cholecystitis (Figure 1). A hepatobiliary iminodiacetic acid (HIDA) scan then ruled out acute cholecystitis (Figure 2). Magnetic resonance cholangiopancreatography (MRCP) was also performed, which was negative for ​​choledocholithiasis. CT of the chest, abdomen, and pelvis with contrast showed bilateral axillary, bilateral hilar, mediastinal, cardiophrenic, upper abdominal, retroperitoneal, and pelvic lymphadenopathy, as well as splenomegaly, which was concerning for lymphoma (Figure 3). A left inguinal lymph node excisional biopsy was performed. Hematology/oncology was consulted and recommended outpatient follow-up of biopsy results. The patient was further discharged with outpatient hematology /oncology follow-up. The results further showed atypical lymphoid hyperplasia that was inconclusive for lymphoma (Figure 4).

**Table 1:** Pertinent laboratory values on initial presentation and on readmission.

Test	Laboratory values on initial presentation	Laboratory values on readmission	Reference Range
White cell count	9.6 K/cmm	10.0 K/cmm	4.8-10.8 K/cmm
Hemoglobin	9.7 g/dL	9.5 g/dL	13.1-15.5 g/dL
Mean corpuscular volume	79 fL	80 fL	80-94 fL
Total bilirubin	1.8 MG/DL	1.1 MG/DL	0.2-1.2 MG/DL
Direct bilirubin	1.0 MG/DL	0.5 MG/DL	0.0-0.5 MG/DL
Aspartate aminotransferase	49 U/L	54 U/L	8-34 U/L
Alanine aminotransferase	74 U/L	34 U/L	6-55 U/L
Alkaline phosphatase	293 U/L	182 U/L	40-150 U/L
Lactate dehydrogenase	526 U/L	781 U/L	125-220 U/L
Reticulocyte count	0.4 %	2.9 %	1-2 %
Ferritin	887 NG/ML	2767 NG/ML	22-275 NG/ML
Haptoglobin	208 MG/DL	185 MG/DL	14-250 MG/DL
Lactic acid	2.8 MMOL/L	2.9 MMOL/L	0.2-2.2 MMOL/L
D-dimer	-	3.84 mg/L(FEU)	<0.5 mg/L(FEU)

**Figure 5:**
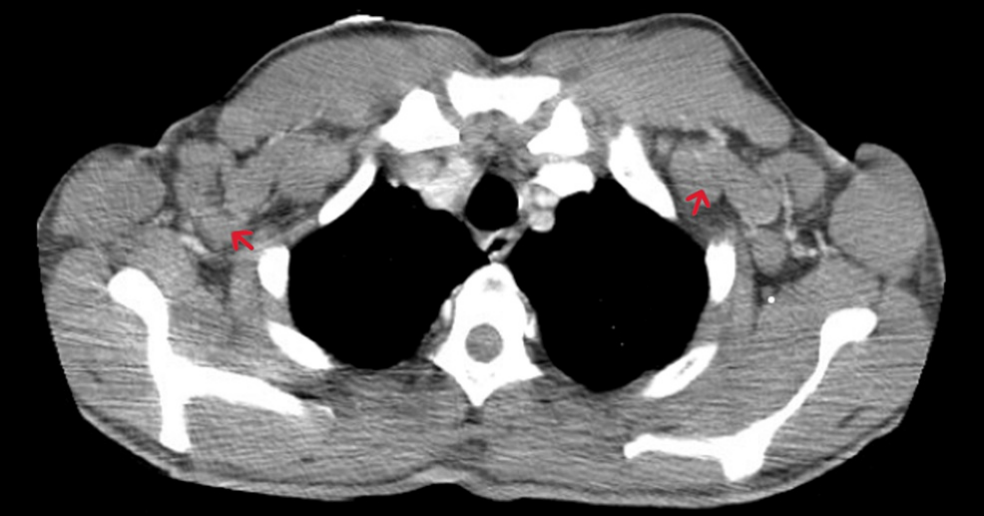
CT angiography (CTA) chest with contrast (axial) showing axillary lymphadenopathy (red arrows).

**Figure 6:**
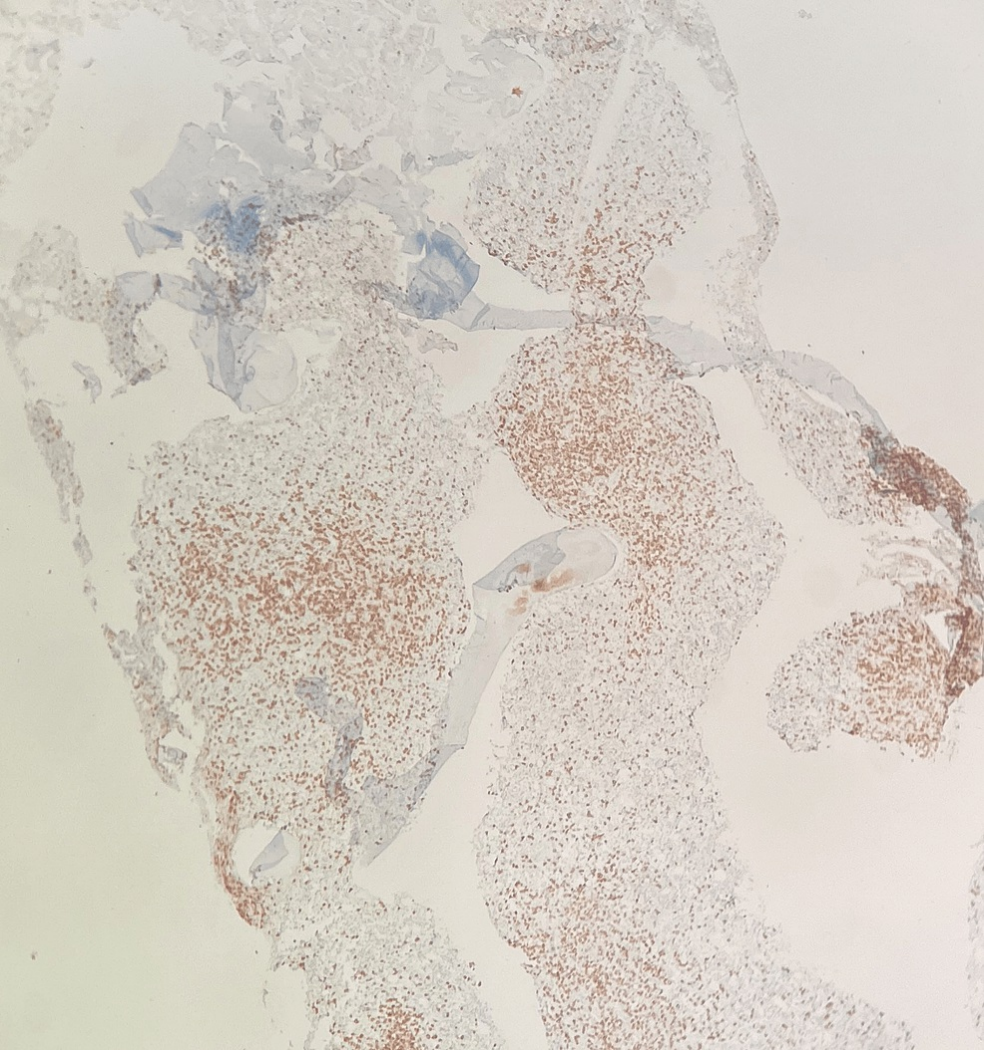
CD3 stain. Bone marrow trephine biopsy under 40x magnification showing sheets of lymphoma cells.

The patient returned to the hospital six days later with similar complaints of fever, chills, and weakness, which had worsened. Vital signs were significant for a fever of 102.4 F, heart rate of 156 beats per minute, blood pressure of 132/71 mmHg, respiratory rate of 21 breaths per minute, and oxygen saturation of 97% on room air. On physical examination, new lymphadenopathy was noted in his neck. His tongue and lips were edematous, and a new diffuse maculopapular rash was present. Laboratory findings on readmission are seen in Table 1. CT angiography (CTA) chest with contrast showed persistent bilateral axillary, bilateral hilar, mediastinal, and cardiophrenic lymphadenopathy (Figure 5). Overnight, the patient acutely decompensated with hypotension, dyspnea, and tachypnea, which required intubation and vasopressor support. He was empirically started on broad-spectrum antibiotics, antivirals, antifungals, and stress dose steroids. This included vancomycin 1750 milligrams (mg) every 12 hours, cefepime 2000 mg every eight hours, doxycycline 100 mg every 12 hours, acyclovir 750 mg every eight hours, micafungin 1000 mg daily, and hydrocortisone 50 mg every eight hours. His hemoglobin and platelets also decreased, requiring transfusions. On peripheral smear, intraerythrocytic inclusions were noted suspicious for parasites, with several increased granulated neutrophils, several hyper-pigmented lymphocytes, and very few schistocytes. Flow cytometry was negative for lymphoma. The patient was weaned off of vasopressors and the ventilator with a follow-up chest X-ray showing a marked clearing of the infiltrate in the lungs. The patient’s rash also started to improve, and steroids began to be tapered. All antivirals, antifungals, and antibiotics except doxycycline, azithromycin, and atovaquone were discontinued as cultures had failed to identify any organisms. All antimicrobials were subsequently discontinued when pathology reported blood smears negative for malaria, Bartonella, and Babesia. Bone marrow biopsy showed lymphoma involvement, but was negative for hemophagocytic lymphohistiocytosis (Figure 6).

Excisional lymph node biopsy pathology that was performed during the first admission was then referred to a hematopathologist at another facility, and a diagnosis of angioimmunoblastic T-cell lymphoma was established. The diagnosis was made by morphology and immunostaining. The specimen represented an enlarged lymph node where the normal architecture is effaced by a paracortical atypical infiltrate of intermediate-size lymphocytes with clear cytoplasm in a background of marked microvascular proliferation. The tumor cells expressed CD2, CD3, CD4, CD5, CD7 (partial, dim) TCR beta, CXCL13 (partial), CD10 (partial) PD-1, ICOS, IDH2 R 172S/T. CD8, CD20, TIM3, LAG 3 were negative in tumor cells. EBER labels scattered small lymphocytes. CD21 and CD23 labels markedly increased follicular dendritic cell meshworks. CD30 labeled 1-2% of all cells. Ki67 staining was high in the neoplastic T-cells (50%). No Hodgkin and Reed-Sternberg cells were detected by the submitted CD30, CD15, OCT-2, Bob 1, and CD45 stains. The submitted ALK1 staining was negative. The patient was transferred to this specialty facility for further management. The patient ultimately received three cycles of cyclophosphamide, doxorubicin hydrochloride (Adriamycin), vincristine (Oncovin), and prednisone (CHOP). While on therapy, he developed respiratory failure secondary to bacterial pneumonia. The patient expired despite best efforts.

## Discussion

AITL is a very rare subset of PTCL, a sub-type of NHL. It has an incidence of 0.05 cases per 100,000 persons in the United States. It is typically seen in the seventh decade of life with a median age of 69 years, with males and females equally affected. A review of the Surveillance, Epidemiology, and End Results (SEER) database from 1973 to 2010 included a total of 1207 cases, of which approximately 79.5% of patients had already progressed to advanced-stage disease on presentation [1]. Based on the data, the 2, 5, and 10-year survival rates were 46.8%, 32.9%, and 21.9%, respectively. AITL is staged according to the Lugano Classification for Non-Hodgkin Lymphoma and is divided into four stages. Stage I is classified as a single affected lymph node region or involving one extra lymphatic region. Stage II involves two lymph node regions on the same side of the diaphragm or localized involvement of an extra lymphatic organ. Stage III is lymph node involvement on both sides of the diaphragm, while stage IV refers to the widespread involvement of one or more extra lymphatic organs such as the lung or bone marrow [2]. The patient presented in this report is consistent with stage IIIB disease. 

Diagnosis is often difficult due to AITL’s vast clinical and histopathologic spectrum that can mimic many reactive and neoplastic processes [3]. Therefore, clinical presentation and symptoms are critical to diagnosis. Patients can present with a systemic illness characterized by B symptoms, which include fevers, night sweats, weight loss, and enlarged lymph nodes [4]. This is similar to the case of our patient who presented with fever, rigors, and palpable inguinal lymphadenopathy. Such symptoms can lead to a broad differential which includes a systemic infection, autoimmune disease, or primary bone marrow (BM) disorder [5]. On physical examination, lymphadenopathy, hepatosplenomegaly, and extranodal involvement are often seen [4]. Up to approximately half of the patients have cutaneous features, and a pruritic morbilliform rash involving the trunk is the most common [6]. As seen in our patient, his rash was also described as maculopapular. Patients also have a characteristic set of lab abnormalities, which may be accompanied by normocytic normochromic anemia, positive direct Coombs test, and an elevated LDH [7]. Diagnosis is also often difficult, and although lymph node biopsy is required to establish the diagnosis of AITL, bone marrow biopsy is frequently performed before obtaining a lymph node specimen [5]. 

CHOP chemotherapy continues to be the gold standard for all PTCL cases. Currently, there is a complete response (CR) rate of 39% in PTCL overall, and 53% CR in AITL [8]. Other studies have shown induction with etoposide followed by CHOP (CHOEP) in PTCL, which has shown promise with a complete response of 51% and an overall response rate (OR) of 82% [9]. Bevacizumab, a monoclonal antibody against vascular endothelial growth factor (VEGF), is currently being investigated as targeted therapy against B-cell proliferation. In a phase 2 study with 39 patients receiving CHOP and Bevacizumab, nine of which completed the planned regimen, a CR of 49%, with a one-year progression-free survival (PFS) of 44%, was seen [10]. However, this regimen was found to cause significant cardiac toxicity and was not explored further [11]. Also, alemtuzumab, a monoclonal antibody against CD-52, can target both B and T-cells that express CD-52. CHOP with alemtuzumab has shown a CR of 71% [12]. Multiple studies using this drug have been done but with significant complications of opportunistic infections in all studies. Such studies support alemtuzumab-CHOP as a potentially effective therapy but with a noted increased risk of Epstein-Barr virus (EBV) and cytomegalovirus (CMV) [12,13]. 

As mentioned previously, our patient with AITL was of Nigerian descent and had also been recently treated for malaria in his home country. In a study of 4125 patients diagnosed with malaria in Sweden from 1987-2015, and who grew up in a malaria endemic-region of Sub-Saharan Africa, it was found that they were at an increased risk for lymphoid neoplasms, in particular, B cell-derived lymphomas [14]. In a study of coinhibitory receptors of T cells, such as CTLA4 and PD1, and their role in infections such as *P. falciparum malaria,* it was noted that flow cytometric analysis showed a more frequent expression of CTLA4 and PD1 on CD4+ T cells of malaria patients than of healthy control subjects. It was also found that acute *P. falciparum *malariainduces *P. falciparum*-specific PD1+CTLA4+CD4+ T-effector cells that coproduce IFNγ and IL-10 [15]. IL-10 is known to be important in regulating the growth and differentiation of cells which is why it plays a significant role in tumors, as well as in inflammation and immune response, and may have played a role in the aggressive nature of disease presented in this case. In a retrospective study of 205 patients who were newly diagnosed with PTCL, it was found that those who had higher levels of IL-10 (≥3.6 pg/ml) in their blood had lower response rates, higher recurrence rates, and lower overall survival (OS) than those with lower IL-10 levels (<3.6 pg/ml) [16]. This was further corroborated in another study where 97 patients with AITL had IL-10 levels evaluated and the high IL-10 group showed inferior OS compared with the low IL-10 group. However, high IFN-γ groups were not significantly associated with OS and PFS [17]. IL-10 levels were not measured in this case but may represent a link between malaria infections and T-cell lymphomas. More investigation is needed to further elucidate any possible connection between malaria and immunosuppression contributing to the presentation. 

## Conclusions

This case of AITL showed many hallmarks seen of the disease with lymphadenopathy, diffuse maculopapular rash, and lab abnormalities, but was challenging to diagnose due to signs and symptoms consistent with an infectious etiology. This emphasizes the difficulty of diagnosing such aggressive types of lymphomas, which can lead to delayed treatment. The effect that malaria and immunosuppression have on AITL also needs to be further explored, as there are limited case reports on this. 
